# Cellulase from *Trichoderma harzianum* interacts with roots and triggers induced systemic resistance to foliar disease in maize

**DOI:** 10.1038/srep35543

**Published:** 2016-11-10

**Authors:** Kandasamy Saravanakumar, Lili Fan, Kehe Fu, Chuanjin Yu, Meng Wang, Hai Xia, Jianan Sun, Yaqian Li, Jie Chen

**Affiliations:** 1 School of Agriculture and Biology, Shanghai Jiao Tong University, Shanghai, P.R. China; 2 State Key Laboratory of Microbial Metabolism, Shanghai Jiao Tong University, Shanghai, P.R. China; 3 Key Laboratory of Urban Agriculture (South), Ministry of Agriculture, Shanghai, P.R. China

## Abstract

*Trichoderma harzianum* is well known to exhibit induced systemic resistance (ISR) to *Curvularia* leaf spot. We previously reported that a C6 zinc finger protein (*Thc6*) is responsible for a major contribution to the ISR to the leaf disease, but the types of effectors and the signals mediated by *Thc6* from *Trichoderma* are unclear. In this work, we demonstrated that two hydrolases, *Thph1* and *Thph2*, from *T. harzianum* were regulated by *Thc6*. Furthermore, an electrophoretic mobility shift assay (EMSA) study revealed that *Thc6* regulated mRNA expression by binding to GGCTAA and GGCTAAA in the promoters of the *Thph1* and *Thph2* genes, respectively. Moreover, the *Thph1* and *Thph2* proteins triggered the transient production of reactive oxygen species (ROS) and elevated the free cytosolic calcium levels in maize leaf. Furthermore, the genes related to the jasmonate/ethylene signaling pathway were up-regulated in the wild-type maize strain. However, the Δ*Thph1*- or Δ*Thph2*-deletion mutants could not activate the immune defense-related genes in maize to protect against leaf disease. Therefore, we conclude that functional *Thph1* and *Thph2* may be required in *T. harzianum* to activate ISR in maize.

Plants are naturally exposed to a range of dangerous and beneficial microorganisms. Efficient strategies to sense danger and rapidly mount defense responses are crucial for plant survival^1^. Plants are resistant to most pathogenic microbes through innate immunity, and the defense mechanism can occur locally at the site of infection and systemically^2^,^3^. Based on the injuries, plants may activate their resistance in two different ways: systemic acquired resistance (SAR) and induced systemic resistance (ISR)^4^. Plants employ SAR to restrict pathogen expansion in systemic tissues by inducing necrosis at the local site upon primary infection^5^,^6^, which is typically characterized by the activation of SA-related genes and pathogenesis-related proteins^7^. Interestingly, ISR is not only initiated by pathogens, but is also induced by root colonization and the interaction with systemic mutualistic or plant growth-promoting microbes (PGPR)^8^.

The coordination of plants and the microbiome occurs during the initial stage of the interactions, in which the signaling molecules play crucial roles and determine the final outcomes of competence^9^. The molecular patterns of plant and microbiome interactions in ISR of plants are described as pathogen- or microbe-associated molecular patterns (PAMPs and MAMPs, respectively)^10^. Several pathogens typically activate the ISR in plants during the interactions with PAMPs^11^. However, not all pathogens are involved in ISR; for example, the necrotrophic pathogen *Botrytis cineria* did not increase the ISR during infection^12^. Upon pathogen infection, the ISR is increased and protects against secondary infections; furthermore, the addition of the *Trichoderma* treatments could accelerate ISR to reduce the infection^13^. Plant cell wall degradation products can be considered ‘microbe-induced molecular patterns’ (MIMPs) that are recognized through receptors such as ‘pathogen-induced modified self’^14^. Thus, the cell wall oligosaccharides originating from plants or pathogenic microorganisms can play an important role in the perception of the invading pathogen by the plant. It is also thought that mimicking pathogen attack using these non-specific elicitors might prove useful in the development of alternative strategies for crop protection, even if the activation of plant defense responses in a non-cultivar-specific manner may not necessarily mediate resistance^15^. A study of the specific microbial elicitors that interact with plant receptors is essential to understand the molecular patterns of the plant-microbe interactions. In this context, our previous study has showed that the C6 zinc finger protein-like elicitor Thc6 induced plant defense responses and provided high levels of systemic resistance against *Curvularia lunata*, a causal agent of *Curvularia* leaf spot in maize^16^, which is widely distributed throughout the world. In the ISR system, defense response signaling is usually able to transfer from the belowground root system to aboveground leaf to protect the plant from foliar disease. However, until now, there has not been substantial research describing those signals and the mechanisms by which they move from the roots to the maize leaf to protect the plant from foliar diseases.

The belowground plant organs of root system that interact with the PGPR might activate sensitive resistance to subsequent pathogen attacks, a phenomenon generally known as ISR^3^,^17^,^18^,^19^,^20^. This response is typically identified through the up-regulation of ethylene (ET)- and jasmonate (JA)-dependent signaling pathways^1^,^4^. The occurrence of PGPR-mediated ISR has been reported in many plant species and is also effective against various pathogens^21^.

In addition to the PGPR, there is another group of root-colonizing *Trichoderma* fungi that have been found to induce plant resistance to pathogens^8^,^17^,^19^,^22^,^23^. Members of the genus *Trichoderma* grow in a wide range of substrates and have been reported as successful biocontrol agents of plant diseases by inducing both local and systemic resistance^19^. Previous studies have reported that the root colonization of *Trichoderma* spp. results in the accumulation of antimicrobial compounds^24^ and several proteinaceous elicitors, such as Sm1^17^ and Epl1^25^, in plant roots. Damage-associated molecular patterns (DAMPs) that are liberated by *Trichoderma* from both plants and fungal preys can be recognized by PRR plant receptors and activate the defense cascades^26^. Nimchuk *et al*.^27^ have described a number of microbial elicitors that induce the plants’ innate defense responses. *Trichoderma* spp. are known to degrade the plant cellulosic biomass by producing hydrolytic enzymes that are collectively called cellulases^28^,^29^. *Trichoderma* cellulase complexes trigger the ISR in plants, such as tobacco, lima bean and corn, by increasing the ET or JA pathways^30^,^31^. This response is a concentration-dependent pattern that occurs based on the *Trichoderma* concentration in the roots and the interaction^32^. However, to date, the mechanism by which the cellulase-like fungal elicitor induces the interaction with plant receptors and its role in ISR are undefined. Therefore, our aim is to identify the role of *Trichoderma* cellulases in the ISR in plants to protect them against invading pathogens.

The present study provides new insights into the mechanisms underlying the process by which the *Trichoderma* and plant root interactions trigger induced systemic resistance against *Curvularia* leaf spot in maize and elicit defense, as well as the characterization of cellulase-like proteins secreted by *T. harzianum*, which are designated *Thph1* and *Thph2*. This study also elucidates the essential roles of *Thph1* and *Thph2* in ISR to *Curvularia* leaf spot in maize using the molecular knock-out (KO) Δ*Thph1* and Δ*Thph2* mutants. In addition, the effects of their colonization on the salicylic acid (SA) and ET content were studied. The effectiveness of the induced resistance against the leaf spot was reported in maize.

## Results

### Screening genes regulated by the transcription factor *Thc6*

**Figure 1:**
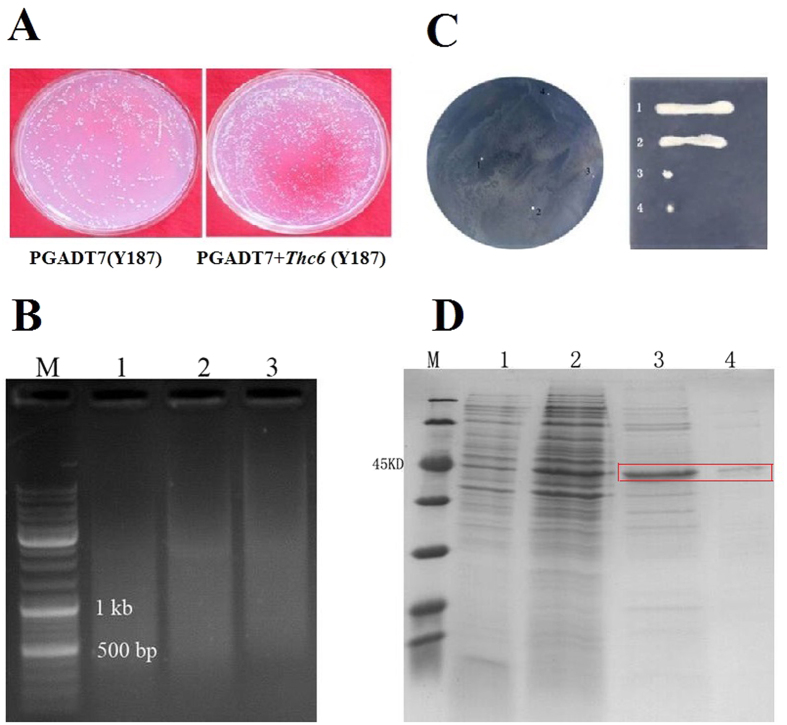
Screening for genes that are regulated by the transcription factor *Thc6*. (**A**) Toxicity of the *Thc6* protein in yeast cells. (**B**) Enzymatically digested genomic DNA. (**C**) Preliminary screening and verification of the interaction of the DNA fragment with the *Thc6* protein. (**D**) Prokaryotic expression of *Thc6* (Lane 1, CK supernatant; Lane 2, CK precipitate; Lane 3, *Thc6* supernatant; Lane 4, Thc6 protein purified by Ni chromatography).

Previously, we showed that a *T. harzianum* C6 zinc finger protein-like elicitor, *Thc6*, induced plant defense responses and provided high levels of systemic resistance against *Curvularia lunata*, a causal agent of *Curvularia* leaf spot, in maize^16^. The yeast strain Y187 carrying a plasmid for expression of Thc6 strain or an empty vector were cultured on yeast peptone dextrose agar (YPDA) media to test toxicity of the Thc6 protein to yeast cells. Expression of Thc6 had no obvious effect on yeast cells. Therefore, this strain was used in subsequent (Fig. 1A). The dispersed bands at 400–800 bp obtained by digestion of genomic DNA with EcoR1/Sac1 (Fig. 1B) were selected for gel extraction. The recovered products were cloned with the linearized pHIS2 carrier, mixed with the corresponding enzymes, and linked with the T4 enzyme overnight at 4 °C. The products were added into the bait vector (PGADT7+*Thc6*) at the ratio of 2:1, co-transformed into competent yeast AH109 cells and transferred the cells to the selective media containing 60 mM 3AT SD/His/Ura/Leu at a dose of 100 μl/dish. Four positive colonies were initially obtained, and two recurring positive clones obtained from single colonies were selected again in lineation. In Fig. 1C, lanes 1 and 2 were the supernatant precipitates in the empty control plasmid. Although IPTG was added to induce the protein, no target protein was observed. Lane 3 was collected from the supernatant of the strain expressing the *Thc6* protein, and it was a distinct protein band selective fragment (SF) underneath the 60-kDa Mark strip. Lane 4 was obtained by Ni column chromatography using solvent (400 mM imidazole) and obtained the target-purified protein (Fig. 1D). Prokaryotic expression of *Thc6* was determined using an electrophoretic mobility shift assay (EMSA).

### Identification of interactive full-length gene amplification products

**Figure 2:**
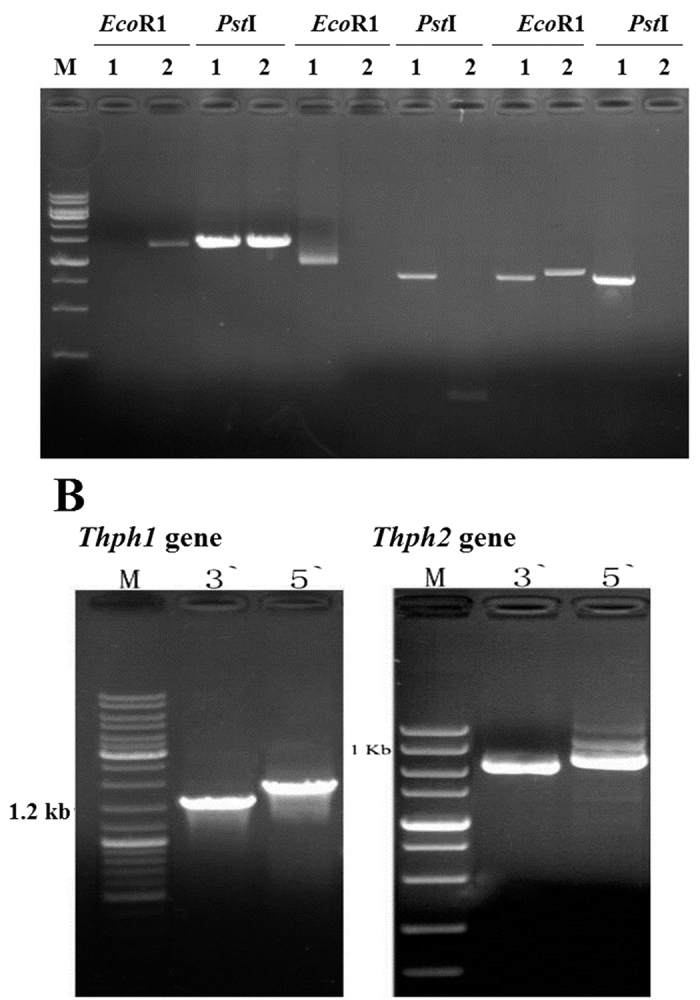
Identification of the full-length *Thph1* and *Thph2* genes. (**A**) Reverse PCR amplification of the sequences flanking the *Thph1*and *Thph2* genes. (**B**) Amplification of the full-length cDNAs of the *Thph1* and *Thph2* genes.

The two positive plasmid clones were sequenced to obtain two target DNA sequences. The two sequences were designed as template primers to amplify the DNA sequences on both sides of the known sequence by inverse PCR (Fig. 2A). Both genes were amplified to obtain approximately 6 Kb of DNA, and the sequence obtained after splicing was verified through PCR amplification. Furthermore, based on the analysis and prediction of the obtained cDNA fragments of two genes, we amplified full-length genes using the 5′ with 3′ RACE method. The genes obtained from the amplification of the full-length cDNAs (Fig. 2B) and subsequent analysis were designated *Thph1* and *Thph2*.

### Amplification of the fragments of the *Thph1* and *Thph2* gene promoters

**Figure 3:**
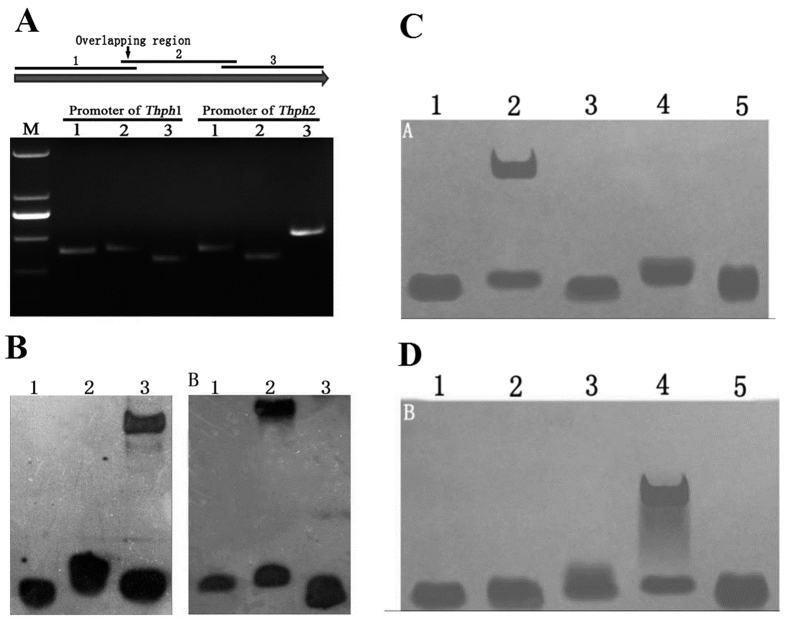
Amplification of the fragments of the *Thph1* and *Thph2* gene promoters. (**A**) PCR amplification of the *Thph1* and *Thph2* promoters for use as probes. (**B**) Interactions of the promoter regions of *Thph1* and *Thph2* with Thc6, as tested by EMSA. (**C**) Interaction of the promoter region of *Thc6* with the *Thph1* promoter. (**D**) Interaction of the promoter region of *Thc6* with the *Thph2* promoter (1–5 promoter fragments).

According to the results of the yeast one-hybrid screen, we selected two sets of ATG gene sequences of 1,139 bp and 1,187 bp. After analysis, the promoter regions of *Thph1* and *Thph2* were divided into three sections, and primers were designed to obtain the target fragments by PCR amplification, followed by purification by labeling the EMSA probes (Fig. 3A). The second segment of the *Thph1* promoter region and the third segment of the *Thph2* promoter region interacted with the *Thc6* protein (Fig. 3B). The *Thph1* and *Thph2* promoters were divided into five segments, and the EMSA was repeated to further verify the interaction between the *Thc6* protein and the *Thph1* and *Thph2* gene promoters. The results showed significant interactions between the second segment of the *Thph1* promoter region and the fourth segment of the *Thph2* promoter region with the *Thc6* protein (Fig. 3C,D). Furthermore, the second segment of the *Thph1* promoter region contained the 5′-GGCTAAA motif, and the fourth segment of the *Thph2* promoter region contained the 5'-GGCTAA motif. The two motifs are a protein-binding site in the promoter.

### Genetic analysis of *Thph1* and *Thph2*

Based on the analysis of the sequence alignments of the proteins encoded by the two NCBI genes, we found that the two genes belong to the family of cellulose hydrolysis enzymes and conserved cellulose binding domains were located in the C-terminus of *Thph1* and N-terminus of *Thph2* (Supplementary Fig. 1). Furthermore, the cluster analysis showed that the *Thph1* protein and *cbh1* protein from *Trichoderma virens* shared the highest homology (82.6% similarity) (Supplementary Fig. 2), whereas the *Thph2* protein and the *cbh2* protein from *Trichoderma reesei* source shared the highest homology (85.7% similarity) (Supplementary Fig. 3).

**Figure 4:**
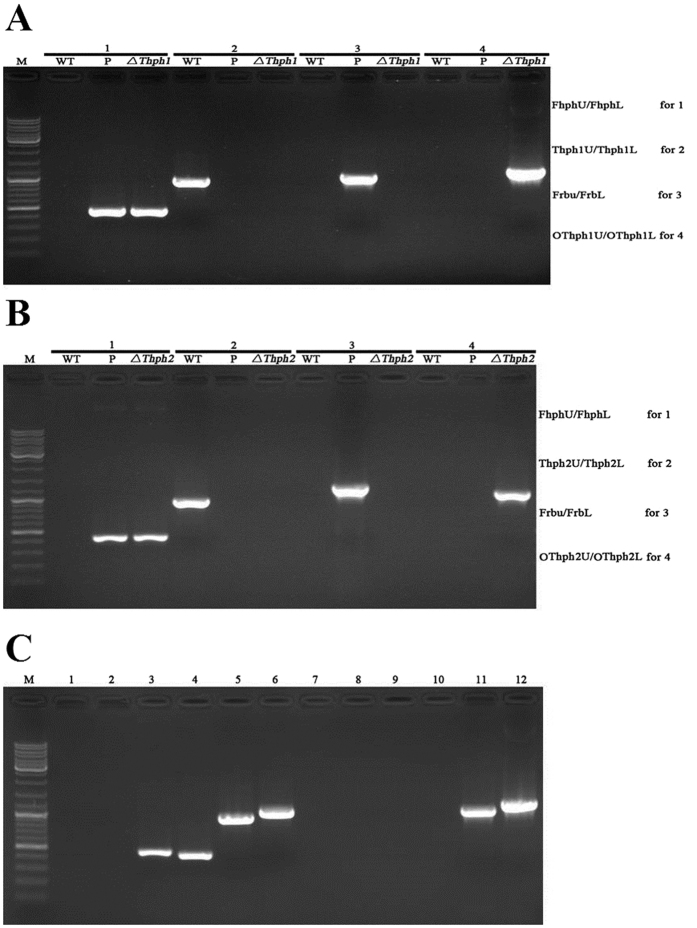
Analysis of the *Thph1* and *Thph2* gene expression levels in the mutant strains. (**A**) PCR confirmation of the *ΔThph1* knock-out mutant using four pairs of primers: Group 1, amplification of the *hph* gene. Group 2, amplification of the *Thph1* gene. Group 3, amplification of right border flanking fragments of T-DNA. Group 4, amplification of the 5′ flanking sequence of *Thph1* (WT, wild-type strain; P, plasmid; *ΔThph1*, knock-out mutant of *ΔThph1*; M, DNA ladder). (**B**) PCR confirmation of the *ΔThph2* knock-out mutant using four pairs of primers: Group 1, amplification of the *hph* gene. Group 2, amplification of the *Thph2* gene. Group 3, amplification of the right border flanking fragments of T-DNA. Group 4, amplification of the 5′ flanking sequence of *Thph2* (WT, wild-type strain; P, Plasmid, *ΔThph2*, knock-out mutant of *ΔThph2*; M, DNA ladder). (**C**) PCR verification of the *ΔThph1*&*Thph2* double knock-out mutant. Lane 1, amplification of the *hph* gene using the WT DNA as the template. Lane 2, amplification of the G418 gene using the WT DNA as the template. Lane 3, amplification of the *hph* gene using the mutant DNA as the template. Lane 4, amplification of the G418 gene using the mutant DNA as the template. Lane 5, amplification of the *Thph1* gene using the WT DNA as the template. Lane 6, amplification of the *Thph2* gene using the WT DNA as the template. Lane 7, amplification of the *Thph1* gene using the mutant DNA as the template. Lane 8, amplification of the *Thph2* gene using the mutant DNA as the template. Lane 9, amplification of the flanking segment of the Thph1 gene using the WT DNA as the template. Lane 10, amplification of the flanking segment of the *Thph2* gene using the WT DNA as the template. Lane 11, amplification of the flanking segment of the *Thph1* gene using the mutant DNA as the template. Lane 12, amplification of the flanking segment of the *Thph2* gene using the mutant DNA as the template.

We analyzed the gene expression from cDNA of KO strains to confirm the successful generation of the mutant stains. The results of the verification of the gene expression indicated that Δ*Thph1* (Fig. 4A), Δ*Thph2* (Fig. 4B) Δ*Thph1* and Δ*Thph2* (Fig. 4C) were successfully produced by *Agrobacterium tumefaciens* Mediated Transformation ATMT homologous replacement.

### The effect of mutant strains *ΔThph1* and *ΔThph2* on cellulose utilization

**Figure 5:**
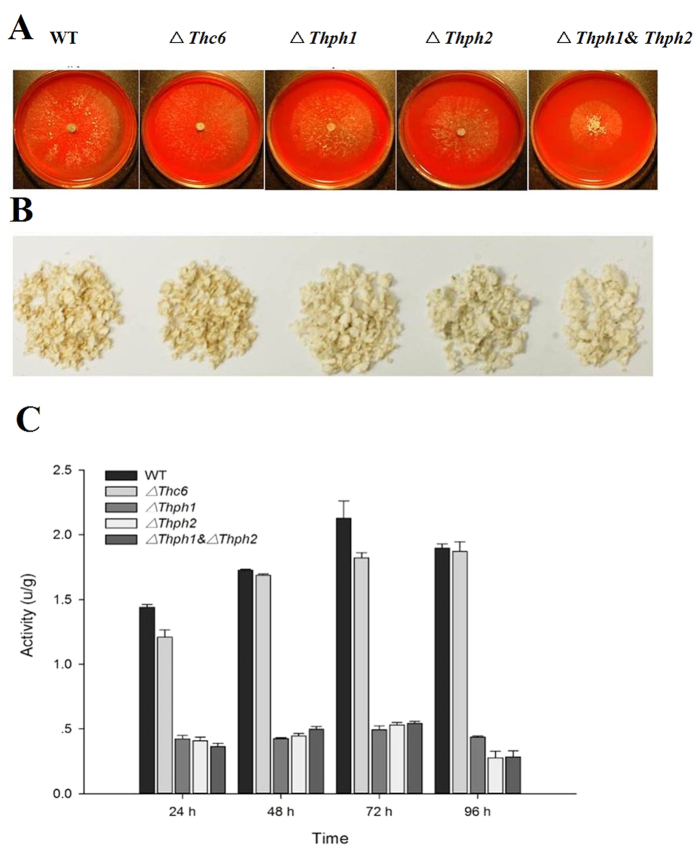
Growth and cellulose utilization of the knock-out mutants and wild strains. (**A**) Growth of the mutant and WT strains on solid medium and (**B**) in liquid medium with cellulose powder as the carbon source. (**C**) Determination of cellulase enzyme activity (WT, wild-type strain; *ΔThc6, Thc6* mutant; *ΔThph1, Thph1* mutant; *ΔThph2, Thph2* mutant*; ΔThph1* & *ΔThph2*, *Thph1* and *Thph2* double mutant).

The effects of the KO strains on cellulose utilization were determined by growing the strains on solid medium and liquid medium that incorporated cellulose powder as the sole carbon source. The *ΔThc6, ΔThph1*, *ΔThph2*, *ΔThph1* and *Thph2* deletion strains did not reveal significant cellulose powder utilization compared with the wild-type (WT) strain. This result indicated that hydrolase genes such as *ΔThc6, ΔThph1*, and *Thph2* had a significant role in cellulose utilization. The capacity of cellulose powder utilization decreased when the hydrolase genes were knocked out of the WT strain and also showed slow growth, particularly when both genes were deleted from the WT strain (Fig. 5A,B).

The cellulose utilization capacity of the WT and mutant strains was further determined by analyzing cellulase enzyme production from the liquid culture using the dinitrosalicylic acid (DNS) method. The results showed that cellulase activity attained a high peak during 72 hours of incubation. The *ΔThc6* mutant strain had no impact on cellulase activity compared with the WT strain, whereas the cellulase activity was significantly decreased in the *ΔThph1*, *ΔThph2*, *Δ*Thph1 and Thph2-KO strains. These results illustrated that these genes were significantly related to cellulose utilization and cellulase enzyme production (Fig. 5C).

### Prokaryotic cellulase expression

**Figure 6:**
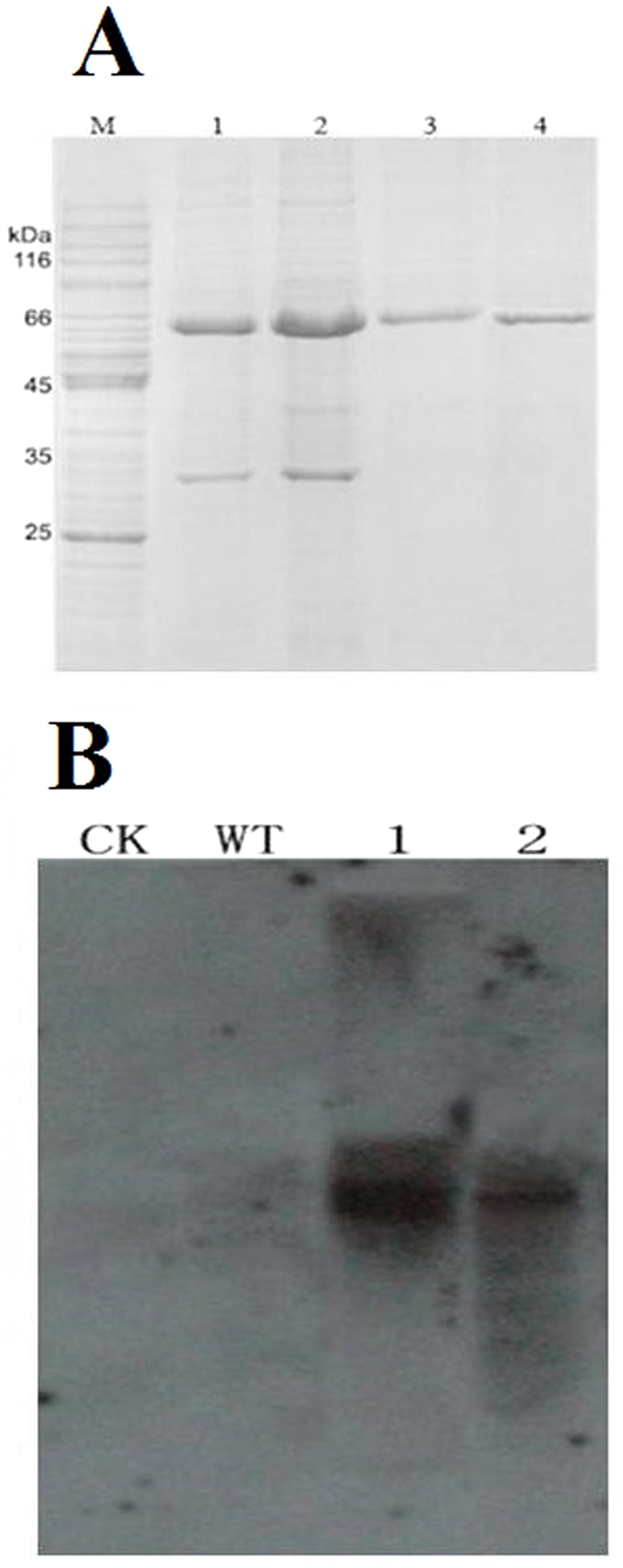
Prokaryotic expression of cellulases. (**A**) Prokaryotic expression of *Thph1* and *Thph2*. Lane M molecular marker; Lane 1, *ΔThph1* supernatant; Lane 2, *ΔThph2* supernatant; Lane 3, Ni-purified *Thph1* protein; Lane 4, Ni-purified *Thph2* protein. (**B**) Detection of the target protein by western blot analysis (CK, 1% carboxymethylcellulose; WT, wild-type strain; 1, mutants containing *Thph1* + His tag; 2, mutants containing *Thph2* + His tag).

The prokaryotic expression of *Thph1* and *Thph2* were analyzed by digesting the open reading frames (ORFs) of *Thph1* and *Thph2*, respectively, and ligated with plasmid pET; 28a-*Thph* was further verified by PCR and DNA sequencing. Then, pET 28a-*Thph* with an N-terminal fusion and metal-binding 6x His tag was transferred into *E. coli* strain BL21 (DE3) to which 1 mM IPTG was added to induce gene expression. A soluble fusion protein was used for the SDS-PAGE and western blot analysis (Fig. 6A). We identified that the recombinant protein bands of the two proteins were approximately 60 kDa, and the pure proteins achieved concentrations of 710 μg/mL and 808 μg/mL.

Maize root tissues were collected at the four-leaf stage, and the proteins were extracted and analyzed using two-dimensional protein electrophoresis and western blotting with an anti-His antibody as the primary antibody to identify the secreted proteins. The results showed that no His-tagged proteins were secreted from the wild or untreated (CK) root samples, but the root samples that were treated with mutant strains *Thph1* (1) or *Thph2* (2) that had integrated the His tag displayed clear bands (Fig. 6B). This result indicated that *Thph1* or *Thph2* proteins were secreted in maize root tissue.

### *Trichoderma* secretes cellulases in the maize root

**Figure 7:**
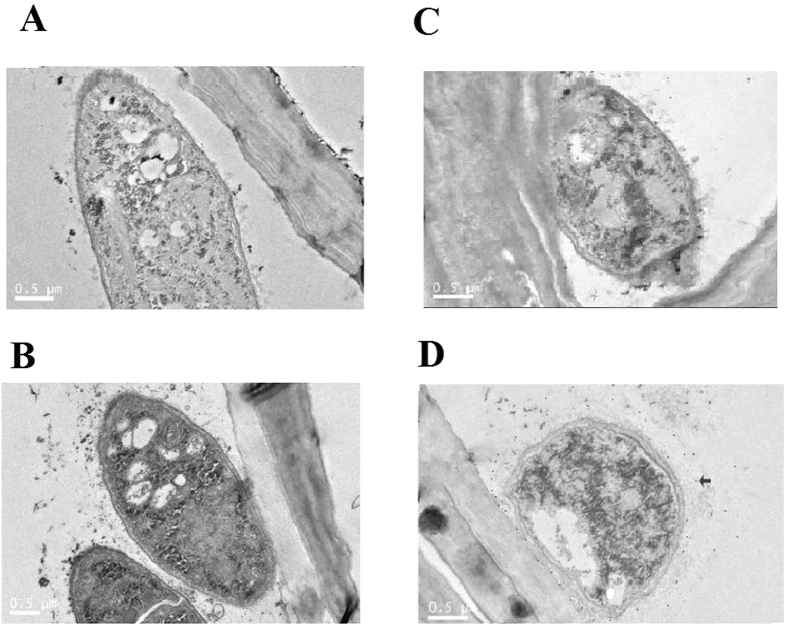
Detection of the target protein by immunoelectron microscopy (Colloidal Gold antibody Conjugates). (**A**) Pretreated with *ΔThph1* but no antibody. (**B**) Pretreated with WT but no antibody. (**C**) Pretreated with *ΔThph1* and antibody. (**D**) Pretreated with WT and antibody. The arrow in D indicates the protein.

The secretion of the *Thph1* cellulase in maize root was further verified by immunogold electron microscopy. Black colloidal gold particles were not detected in the maize root tissue that was treated with the *ΔThph1*-KO mutant in the absence (Fig. 7A) or presence of antibody (Fig. 7C) or in the WT strain without antibody (Fig. 7B), but this cellulase was observed in the WT *T. harzianum*-treated strain (Fig. 7D). These results revealed that the *Thph1* protein was significantly secreted into the maize roots.

### Effects of the *Trichoderma* mutants and WT strains on root colonization, activation of reactive oxygen species production, and lesion inhibition in maize

**Figure 8:**
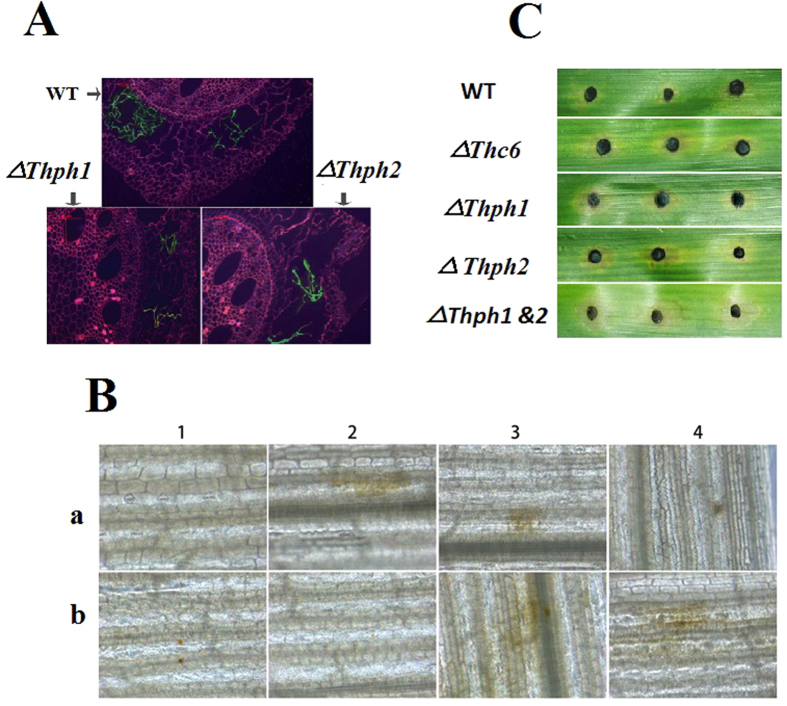
Effect of the *Thph1* and *Thph2* genes on *Trichoderma* colonization, reactive oxygen species production and lesion inhibition in maize. (**A**) Colonization of the *ΔThph1*, *ΔThph2* and WT strains of *Trichoderma* in maize root (WT, wild-type strain with the eGFP tag; *ΔThph1, Thph1* knock-out (KO) mutants with the eGFP tag; *ΔThph2, Thph2* KO mutants with the eGFP tag). (**B**) Changes in the reactive oxygen species (ROS) levels in maize (A1, blank control; A2, pretreated with WT; A3, pretreated with *ΔThc6;* A4, pretreated with *ΔThph1*; B1, pretreated with *ΔThph2*; B2, pretreated with *ΔThph1 & ΔThph2*; B3, pretreated with WT+*Thph1* protein; B4, pretreated with WT + *Thph2* protein. (**C**) Anti-pathogenic effect of the treatments with the different mutant and WT strains of *Trichoderma* on inhibiting lesions in maize (WT, wild-type strain; *ΔThc6, Thc6* mutant; *ΔThph1, Thph1* mutant; *ΔThph2, Thph2* mutant*; ΔThph1* & *ΔThph2*, *Thph1* and *Thph2* double mutant).

We hypothesized that the expression of the *T. harzianum*-derived *Thph1* and *Thph2* genes in maize root depends on its ability to colonize maize roots. Therefore, we tested the colonization of green fluorescent protein (eGFP)-tagged *ΔThph1*- and *ΔThph2*-KO mutants and the WT strain using the ATMT method. The results showed that the colonization of *ΔThph1* or *ΔThph2* in the corn root epidermis was significantly reduced compared with the WT strain of *Trichoderma* (Fig. 8A). In addition, the colonization of the *Trichoderma* mutant and WT strains in maize root was quantified by RT-qPCR. The calculated log_10_ of the *Trichoderma* DNA concentration ranged between 0.27–1.59 ng.g^-1^ in maize root (Supplementary Fig. 4) and it was higher in the WT strain and lower in the *ΔThph1*&*2* strain. These observations of *Trichoderma* colonization in maize root by microscopy and the RT-qPCR assay confirm that disruption of the *Thph1* and *Thph2* genes in *T. harzianum* could influence its colonization in maize.

The determination of the reactive oxygen species levels (ROS) in maize revealed that the mutant strains had a significant influence on ROS generation. The results indicated that the untreated maize (CK), single- or double-KO strains did not show ROS production, whereas the WT, *ΔThc6*, WT+Thph1 (protein), and WT+Thph 2 (protein) strains showed significant ROS production, which is indicated as brown plaques (Fig. 8B). Therefore, the KO of these two genes in *T. harzianum* could affect the maize-based expression of ROS.

The lesion areas were determined in maize leaves that were infected with *C. lunata* and treated with the mutant and WT strains to further validate the impact of the *T. harzianum Thc6*, *Thph1*, and *Thph2* genes on maize defense against *Curvularia* leaf spot. The results indicated that the lesion area was increased in maize leaves that were treated with *ΔThc6*, or the single or double mutants of *ΔThph1* or *ΔThph2*, whereas the lesion area was reduced in the treated WT strain (Fig. 8C). These results indicated that these genes had a significant impact on the disease resistance of corn leaves.

### Function of cellobiose released from *Trichoderma*-colonized roots in induced systemic resistance

**Figure 9:**
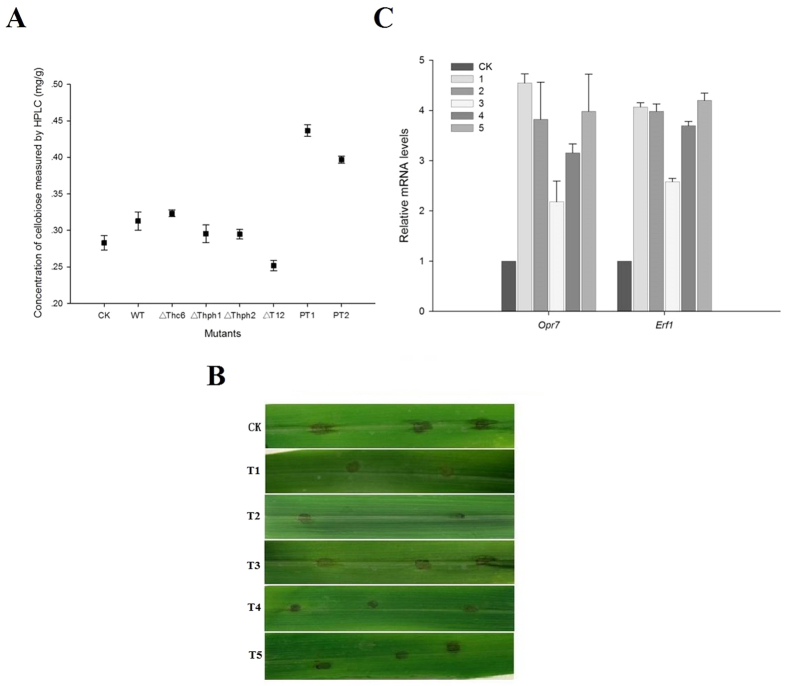
Effects of the different mutant and WT *Trichoderma* strains on (**A**) the cellobiose concentrations in maize root (CK, untreated maize root; WT, wild-type strain-treated maize root; *ΔThc6*, mutant *ΔThc6*-treated maize root; *ΔThph1*, mutant *ΔThph1*-treated maize root; *ΔThph2*, Mutant *Thph2*-treated maize root; *ΔT12*, double knock-out mutant-treated maize root; PT1, WT + *Thph1* protein-treated maize root; PT2, WT + *Thph2* protein-treated maize root). (**B**) Cellulase- or cellobiose-induced systemic resistance in maize. C. qRT-PCR analysis of defense-related gene (Opr7 and Erf1) expression levels in maize leaves that were pretreated with Cellulase+cellobiose (CK, untreated; sample no. 1, Cellulase (10 μg/ml); 2, cellobiose; 3, heat-inactivated cellulose; 4, Cellulase+cellobiose, 5, heat-inactivated cellulase+cellobiose).

Previous studies have found that cellulase functions to induce resistance; the next question is whether a cellulase enzyme is directly involved in the induction of resistance or indirectly through its degradation of cellobiose in the root tissue cell walls and release of induced resistance factors. Therefore, we determined cellobiose production in maize root using HPLC (Fig. 9A and Supplementary Table 1). The results indicated that the amount of cellobiose produced was reduced in maize roots that were treated with the *ΔThph1*- or *ΔThph2*-KO mutant strains compared with the treated WT strain (Fig. 9A and Supplementary Table 1). Moreover, the cellobiose content was significantly increased in maize roots that were treated with the *Thph1* or *Thph2* proteins compared with the treated WT strain. The binding assays for cellulase activity previously illustrated that the two proteins were involved in the degradation of cellulose. The degradation of plant cell wall polysaccharides induces plant defense responses^33^,^34^.

The results revealed the effect of cellulase or cellobiose treatments of maize lesions on inducing resistance and showed a diminished capacity after the enzyme was inactivated (Fig. 9B). The qRT-PCR assay showed that cellobiose had a significant impact on the expression of all corn root defense-related genes (Fig. 9C). The corn roots from the WT strain that were treated with cellulose and cellobiose indicated that the two substances could enhance the induced resistance ability of the WT maize.

### Expression of maize defense genes during *T. harzianum*-induced resistance to *C. lunata*

Several reports denote that the expression of defense-related gene is dependent on the *Trichoderma* concentration and incubation time. Therefore, we analyzed the relative mRNA expression of maize defense genes (Opr7, Pr4, Aoc1, and Erf1) in samples collected from the WT *Trichoderma*-treated maize on different days to elucidate the potential involvement of the JA/ET signaling pathway in the induced resistance of maize triggered by *T. harzianum*. The statistical analysis of the obtained results indicated that the *Trichoderma* DNA concentrations and relative mRNA expression levels of Opr7, Pr4, Aoc1, and Erf1 were significantly increased as the number of days of the *Trichoderma* treatments increased (Supplementary Table 2). The subsequent correlation analysis indicated that the relative mRNA expression levels of Opr7, Pr4, Aoc1, and Erf1 positively correlated with the *Trichoderma* DNA concentrations (Supplementary Table 3). Furthermore, we studied the effects of the mutant and WT strains on maize defense gene expression. The relative mRNA expression levels of Opr7, Pr4, Aoc1, and Erf1 in maize leaves and roots showed significant variations among the treatments with the *Trichoderma* WT and/or mutant strains (Supplementary Figs 5 and 6). The treatments with the *Thph1* or *Thph2* proteins significantly increased the defense-related mRNA expression levels in maize.

We analyzed the JA content in the KO mutants, the WT strain and protein-treated maize leaves or root by LC-MS to further analyze the defense response. The JA content of the maize leaves was higher in the protein-treated plants than in the treated wild strain (Supplementary Fig. 7). These results indicate that the protein content in the leaves could increase the JA content. However, no obvious differences were observed with the pretreatments with the different mutants, which might be related to the induction time and the specific mechanism. A similar pattern was observed for the JA content in roots (Supplementary Fig. 7a). The effect of each mutant treatment was not obvious, but the *Thph1* protein treatment significantly increased the JA content.

Furthermore, we analyzed the effects of the WT strain and KO mutants on the root weights, shoot height of maize, and lesion size in maize infected with *C. lunata*. The results showed that root weights, shoot height of maize, and lesion size varied significantly between the treatments with the different mutant and WT strains. The root weight was increased in the maize treated with the WT strain compared with the plants treated with the KO strains (Supplementary Fig. 8A). The shoot height of the maize was increased in the untreated maize and WT strain-treated maize compared with the maize treated with the KO strains (Supplementary Figure 8B). The lesion area size showed an increasing trend from WT to *ΔThc6*, *ΔThph1* and *ΔThph2*; however, the lesion was comparatively reduced in the maize treated with the WT strain compared with the maize treated with the mutants (Supplementary Fig. 8C).

### Global exploration of the regulation of defense-related proteins in maize leaves by *Trichoderma Thph1* and *Thph2*

**Figure 10:**
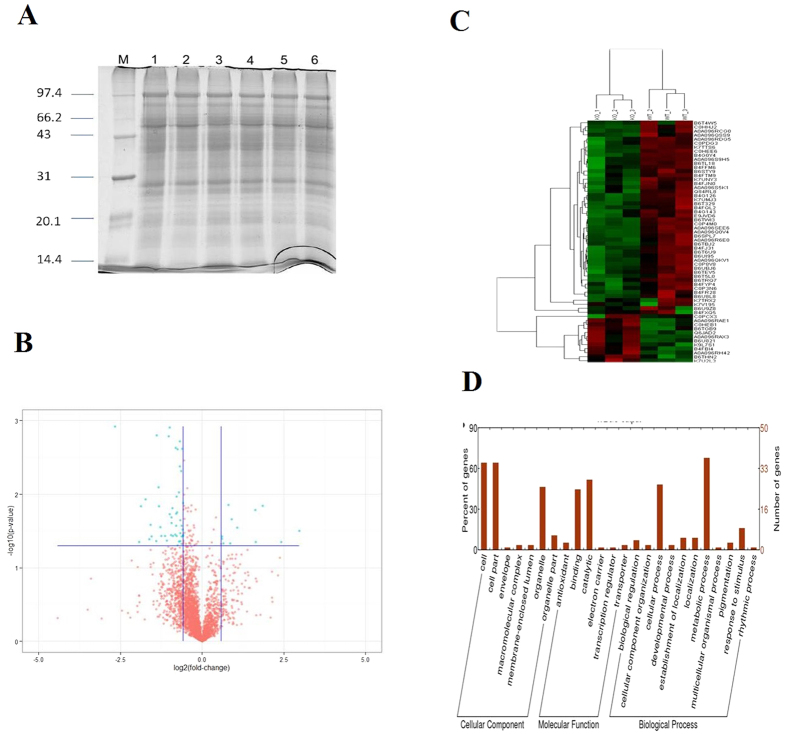
Global exploration of defense-related proteins in leaf that were up-regulated by *Trichoderma ΔThph1* and *ΔThph2*. (**A**) Proteins extracted from the samples: lanes 1–3, WT; lanes 4–5, *ΔThph1*. (**B**) Blue dots in the volcano plot of the protein expression levels indicated the differences in the proteins. (**C**) Thermal clustering analysis. (**D**) Categorical analysis of the proteins.

The proteins that were extracted from the maize leaves that were treated with the WT strain or KO Δ*Thph1* showed that the protein quality was suitable for the subsequent experiments (Fig. 10A, Supplementary Table 4). The homogenized sample was quantitatively identified by mass spectrometry and provided 18,019 peptides from 3,271 protein sequences. The differentially expressed proteins were screened in the subsequent analysis based on the ratio of WT/Δ*Thph1* (>1.5/<0.667). All the proteins were divided into three categories and 21 subcategories (Fig. 10B,C). The analytical results revealed that among the total of 43 proteins, 14 proteins were downregulated, and 12 proteins were up-regulated, and the responsible genes identified in this study are depicted in Fig. 10D and Supplementary Tables 5 and 6. Therefore, the comprehensive results showed that the extracellular cellulase genes *Thph1* and *Thph2* played significant roles in the *T. harzianum* and maize interaction, as well as their ability to control the corn defense response.

## Discussion

### *Trichoderma harzianum* induces systemic resistance in maize

The present study was adapted to use the ATMT method to analyze various mutants of the *Thph1* and *Thph2* genes, as well as the prokaryotic expression of the protein products of these two genes. The cellobiose content in roots was measured by HPLC, and the qRT-PCR analysis of these two genes indicated that they positively correlated with the degradation of cellulose and were involved in the regulation of the *Trichoderma*-mediated induced resistance response in corn. Immunoelectron microscopy also indicated that the *Thph1* protein was involved in *T. harzianum* colonization in maize root tissue. The qRT-PCR analysis illustrated that cellulase or cellobiose had the ability to induce defense responses in maize leaves. In the present study, the label-free quantitative proteomics analysis revealed that the deletion of *Thph1* in *T. harzianum* impacted the expression levels of 12 proteins in maize leaves. The proteins identified in this study included MYC2, a transcription factor and a key gene involved in the JA signaling pathway. In addition, the expression levels of the ACO protein in the ET pathway showed that ACO is involved in ethylene synthesis in plants. The expression level of peroxidase showed that it is involved in ROS degradation in plants.

### *Trichoderma* cellulase secretion is involved in the systemic induction of maize resistance to leaf spot

The cellulase-like proteins *Thph1* and *Thph2* play essential roles in the specificity, recognition, and adhesion of certain symbiotic fungi^30^,^35^. The known function of fungal cellulases is to degrade cellulose-rich plant cell walls, and these enzymes are being studied for their role in conversion of biomass into energy, in order to provide sustainable solutions to the energy problem. Only a few studies have reported the basic description of cellulose-induced defense^16^, but there is currently no research on the mechanism of cellulose-induced plant defense. *Trichoderma reesei* cellulases can cause a series of defense reactions in tobacco leaves, such as the cytoplasmic contraction, nuclear accumulation, and ROS production^35^. The leaves can be induced to secrete ethylene in the *Trichoderma viride* cellulose-treated tobacco (*N. plumbaginifolia*), soybean (*Phaseolus lunatus*), or maize (*Zea mays*)^30^. In the present study, the molecular mechanism of foliar resistance against maize spot was elucidated, and the results showed that the roots interacted with cellulases secreted from *T. harzianum*. However, the cellular regulatory mechanisms by which the *Trichoderma* cellulase genes induce defense reactions in maize are yet to be analyzed.

The present study reports the purification and functional characterization of *Thph1* and *Thph2*, proteins isolated from culture filtrate of *T. harzianum*. The genes were named *Thph1* and *Thph2* after the two full-length cDNA genes were obtained and subsequently analyzed.

### The defense response regulation network was systemically induced by *Trichoderma Thph1* and *Thph2*

Genes of the *Thph* gene family mediate enzymatic hydrolysis of cellulose and play positive roles in regulating plant resistance to pathogens. The up-regulated genes that were identified according to their spots are described (Supplementary Tables 5 and 6). The present study showed that the MYC2 protein was regulated in induced reaction transduced in the plant JA pathway and increased the regulation of the plant defense response^36^,^37^. The chlorophyll protein/protochlorophyllide reductase regulates chlorophyll biosynthesis and catalyzes the protochlorophyllide ester reduction of chlorophyll esters in plants^38^. The proline-rich precursor is a plant cell wall constituent involved in the formation of the cytoskeleton^39^. The 1-amino-6 protein cyclopropane-1-carboxylic acid oxidase (ACO) is involved in ethylene biosynthesis in variety of plants (apples, bananas, peaches, and tomatoes)^40^,^41^,^42^. The ACO gene was successfully cloned, and its expression was induced by external stimuli, such as mechanical damage, pathogen infection, stress and a variety of hormones in plants (such as ethylene, ABA, and IAA). Therefore, the expression level of the ACO gene in plants can be used to measure the ethylene content.

Plant peroxidase (POD) is involved in catalyzing hydrogen peroxide oxidation reactions^43^,^44^. POD participates in the degradation of auxin, which is involved in GSH and NADH catalysis, tyrosine oxidation and ROS degradation^45^. The ferredoxin protein has been reported in many plants and plastid enzymes are derived from electronic ferredoxin, including sulfur reductase, fatty acid desaturase, nitrogen reductase and thioredoxin reductase^46^. Pyridoxine phosphate oxidase is involved in the de novo synthesis of vitamin B6 in plants in one oxidase pathway, which catalyzes the phosphorylation of pyridoxine (PNP) and then pyridoxamine phosphate (PMP) is oxidized to pyridoxal phosphate (PLP)^47^. Molecular chaperones are members of a large family of proteins in cells^48^. The heat shock protein is the largest of a class of chaperone proteins^49^. This protein has a variety of biological functions, such as assisting in the folding, assembly, transport and degradation of biological macromolecules involved in regulating the cell cycle, anti-aging activity, and apoptosis^50^.

### Induced systemic resistance model in maize

**Figure 11:**
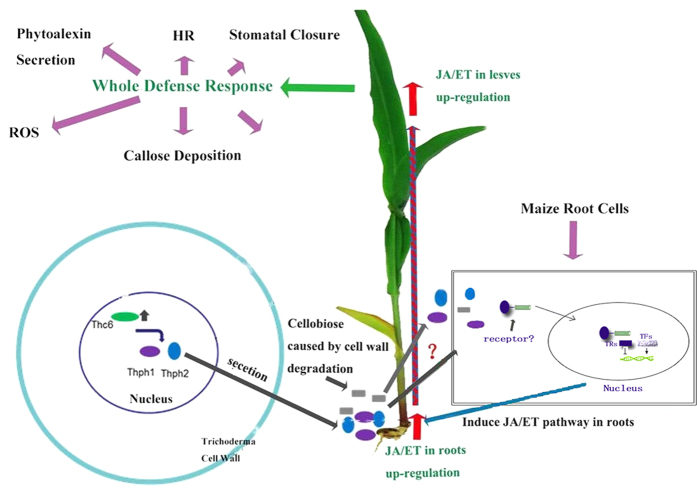
The regulation of the induced systemic resistance-related gene response network in maize. Model of the induced systemic resistance of maize regulated by the *Thc6, Thph1* and *Thph2* genes of *Trichoderma harzianum*.

MAMPs and DAMPs have been widely viewed as the basic model of plant immunity induced by biocontrol agents^51^. In our case, we demonstrated that two types of cellulases (exo-1,4-β-D-glucanase and endo-1,4-β-D-glucanase) functioned as effectors to initiate the MAMP response, and cellobiose acted as an effector to induce the DAMP response; both effectors might work together in the induced systemic resistance in maize against leaf diseases. Based on our data, the interaction of maize root and *Trichoderma* might activate the transcription factor *Thc6*, which then positively regulated the expression of the *Trichoderma* cellulase genes *Thph1* and *Thph2*. The secretion of cellulases from *T. harzianum* might prime the maize root defense system and then the leaf defense responses were activated (MAMPs) by the long distance transduction of defense signals. The cellobiose released from the degraded maize root tissue by *Trichoderma* cellulase was further used to strengthen the ISR effects against leaf spot (DAMPs)^26^. With the activation of the maize root defense system, the root fungal infection stimulates JA/ET signal transduction through phytoalexin accumulation in leaves, thereby increasing *Curvularia* leaf spot disease resistance in maize leaves (Fig. 11).

The following features of this model still need to be clarified: (1) the expression of the transcription factor *Thc6* is not highly relevant to cellulose degradation, indicating there might be some regulatory activity in the *Thc6* repressor; (2) it is still unclear in what way *Trichoderma*-sourced cellulase and maize-sourced cellobiose cooperate in the process of ISR against foliar diseases in maize; (3) the recognition sites of cellulase and cellobiose on maize root need to be identified, in other words, the receptors (Pattern Recognition Receptor, PRR) of cellulase and cellobiose on root must be determined; and (4) *Trichoderma* is stimulated when it interacted with maize leaves and increased the production of JA and ET signaling substance from the maize leaves; alternatively, some unknown spread anti-factor might induce JA and ET signaling in leaf blade. This study only demonstrates that *Thc6*, *Thph1* and *Thph2* were induced in maize (Inbred line Huangzao 4) to protect against leaf spot, but it is not clear whether this mechanism has undergone selection to promote plant defense against other spot diseases, and whether the model applies to other maize varieties.

## Materials and Methods

### Plasmids, fungal strains and plant materials

The beneficial fungal strain *Trichoderma harzianum Th22* and the pathogenic strain *Curvularia lunata* CX3 were used in this study. The plasmid pCAMBIA1300 and *Agrobacterium tumefaciens* strain AGL1 (a T-DNA donor) were kindly provided by Dr. Chu Long Zhang (Institute of Biotechnology, Zhejiang University in Hangzhou, P.R. China). The strains were routinely maintained on PDA (Difco Laboratories, Detroit, Mich). A Thermo light shift chemiluminescent EMSA kit 20148 and probe labeling kit 89818 were used for EMSA experiments to screen the promoter region. *S. cerevisiae* Y187 strain and single hybrid vector pHIS2 plasmid were used for the yeast one-hybrid assay. The maize (Inbred line Huangzao 4) seeds were used in this study were kindly provided by Dr. Wang (Academies of Agricultural Sciences in Shenyang, China).

### Constructing the carrier by ATMT

The Camv35S promoter and *hph* ORF were excised, and the fragment was purified. The resulting plasmid was pC1300-h. Subsequently, a 1,345-bp fragment containing the *hph* ORF (1,026 bp), and promoter region (319 bp) was amplified from plasmid 1003 with the HiFi polymerase using primers TkhU (upper) and TkhL (lower). An *Eco*RI site and *Xho*I site were added to the upper primer and lower primer, respectively. After digestion with the appropriate restriction enzymes, the fragment was gel purified and inserted into *Xba*I/*Bam*HI-digested pC1300-h to produce plasmid pC1300th^52^. The gene KO plasmid pC1300kh was also constructed^52^.

### Yeast one-hybrid assay

We applied a yeast one-hybrid assay according to the instructions of Matchmaker^®^ Yeast One-Hybrid Library Screening System User Manual (Clontech Laboratories, Inc., A Takara Bio Company (US)) to study the interactions of the protein and DNA sequences. We prepared yeast AH109 cells to generate the prey vectors for the yeast one-hybrid assay. The multiple cloning site (MCS) of the bait vector (pGADT7 AD; Supplementary Fig. 9) was digested with *Eco*RI/*Bam*HI and the Thc6 ORF was inserted by PCR; in contrast, the isolated target bait sequence (*Thc6)* was inserted in the bait vector (pGADT7 AD) using T4 ligase. The promoter DNA fragment was digested and linked to the pHIS2 carrier (Supplementary Fig. 10). Successful transformations of the bait and prey vectors were verified by PCR screening and analyzing the vector sequences. After we constructed a suitable BD vector containing the gene in competent yeast Y187+Thc6, the obtained transformants were subjected to resistance analysis by growing them in different yeast growth media; the empty vector was used as a control.

### Cloning of the flanking sequence and detecting the T-DNA insert

Reverse PCR was performed to clone the flanking sequence of the T-DNA insert. The genomic DNA of the mutants was digested with an appropriate single enzyme and collected by absolute ethyl alcohol precipitation. The digested DNA was self-linked by ligation with the T4 enzyme overnight at 4 °C. After precipitation with absolute ethyl alcohol, the ligation products were used as templates for PCR amplification.

### Detecting the interaction between *Thc6* and the promoters of the two genes

Full-length Thc6 was cloned as described in a previous study^16^. The promoter of the Thc6 gene was identified using EMSA. Briefly, IPTG was added to the culture medium at a final concentration of 0.5 mM to induce the production of the exogenous protein in *E. coli* at 24 °C for 12 h. The collected cells and purified exogenous protein were detected by SDS-PAGE and the interactions between the *Thph1* and *Thph2* promoter regions with Thc6 were tested by EMSA (Thermo Light Shift chemiluminescent EMSA kit 20148). Competent yeast samples were prepared after centrifugation at 1000 × *g* for 5 min and were resuspended in an NaCl (0.9%) solution. The genomic DNA of the wild-type (WT) strain was digested with three different dual enzymes, mixed with the pHIS2 carrier that had been digested with the corresponding enzymes, and ligated with the T4 enzyme overnight at 4 °C. A yeast one-hybrid kit (Matchmaker^TM^ One-Hybrid library construction and screening kit) was used.

### Isolation of *Thph1* and *Thph2* and quantitative real-time polymerase chain reaction (RT-qPCR)

DNA was extracted using the standard cetyltrimethylammonium bromide (CTAB) protocol. For the Southern blot analysis, the DNA was digested with restriction enzymes and separated on a 0.9% agarose gel. The DNA gel blot, probe labeling reactions and hybridization were performed using a Gene Images CDP-Star Detection Kit (RPN 3680, GE Healthcare). The expression of the *Thph1* and *Thph2* genes was studied using EMSA and PCR assays.

### Construction of the *Thph1* and *Thph2* deletion vectors

A 2,249-bp promoter and the *Thph1* and *Thph2* ORFs were digested with *Kpn*I and inserted into the Kpn1-digested pC1300N vector to obtain the flanking regions of the *Thph1* and *Thph2* ORFs to construct the deletion strains^52^. A homologous recombination caste was constructed, and the flanking sequences of *Thph1* and *Thph2* were cloned as ko-up-F/Ko-up-R (5′ region) and Ko-down-F/Ko-up-R (3′ region) to generate the *Thph1*- and *Thph2*-deletion transformants. The cloned flanking sequences were digested with *Hin*dIII, *Xba*I, *Kpn*I, or *Sac*I (Fermentas, Canada). *Agrobacterium tumefaciens*-mediated transformation was performed according to the method described by Fu *et al*.^52^.

### Transformation and screening of transformants

Suitable prototropic transformants were selected by consecutive transfer of single colonies to PDA. PCR was used to screen the potential deletion transformants. (1) The *hyg* gene (for hygromycin B resistance) was amplified with primers Hyg-F/Hyg-R, (2) the *Thph1* and *Thph2* deletions were verified by amplifying the *Thph1* and *Thph2* genes, (3) which were used to amplify the targeted gene PCR product from the genomic DNAs from the WT and transformant strains and to identify the *Thph1*- and *Thph2*-deleted transformants. The PCR amplification conditions included 35 cycles (each cycle: 20 s for 95 °C, 30 s at 55 °C, and 20 s at 72 °C). The strains that did not yield PCR products were confirmed as *Thph1*- and *Thph2*-KO strains.

### Analysis of cellulose utilization in the transformants

Cellulose utilization by cultures of selected transformants was compared with the wild-type strain in agar medium, liquid cultures and in cultures where cellulose was the sole carbon source^53^. The culture medium was supplemented with 2% cellulose powder (Sigma, CAS9004–34–6) as the carbon source. Cellulose was added to induce the expression of cellulase. Five hundred milliliters of the WT and transformant strains were inoculated with 1 × 10^7^ conidia in shaker flasks and incubated in a rotary shaker at 28 °C and 180 rpm for 6 days. Then, the mycelia were harvested on filter paper and measured after the preparations were dried at 80 °C for 24 h.

In addition, the activity of the cellulase enzyme was measured in the transformant strains and WT strain culture filtrate using the DNS method. Briefly, 5 ml of the fermented residue were suspended in 150 ml of distilled water and shaken at 120 rpm for 2 h. Then, the filtrate was centrifuged at 10,000 rpm in a high-speed centrifuge for 15 min, and the supernatant was used for the enzyme assay.

Cellulase activity was determined in a reaction mixture (2.0 ml) containing 0.2 ml of the enzymes solution and 1.8 ml of a 1% (w/v) carboxymethylcellulose (CM-cellulose) solution prepared in sodium acetate buffer (50 mM, pH 5.0). The reaction mixture was incubated at 50 °C for 30 min, and the reducing sugar liberated in the reaction mixture was measured using the dinitrosalicylic acid (DNS) method^54^. The reaction was stopped by adding 1.0 ml of 1.0 M sodium carbonate solution. Color development was measured at 405 nm using a plate reader. One unit (U) of enzyme activity was defined as the amount of enzyme required to liberate one mole of glucose per minute under the assay conditions. The enzyme activity is expressed as U.g^−1^ (units per gram).

### *Trichoderma harzianum* root colonization studies

Maize seeds were sterilized in a 10% hypochloride solution for 20 min, soaked in 70% ethanol for 30 s, and then treated with a 1% sodium carboxymethylcellulose solution; finally, *Trichoderma* conidia were added to the solution (1× 10^6^ conidia ml^−1^). After a 12-h inoculation at 25 °C in liquid culture, the seeds were transferred to a Petri dish (containing a filter paper bed with water) and allowed to germinate for 12 h at 25 °C in light culture. An aperture disk (32 holes, 125 cm^3^ hole^−1^, 8 holes per treatments) with nutritive soil was added and then the seeds were grown in a growth chamber at 25 °C for 15 days (four-five-leaf stage). For the secondary screen, the leaf infection assay was performed*. C. lunata* conidia were diluted to 2 × 10^5^ conidia per maize leaf. The infected maize was then grown in a chamber under dark conditions and constant humidity for 48 h to allow the disease to develop.

### Construction of the GFP-tagged *Trichoderma harzianum* strain

*T. harzianum* and KO strains expressing e*GFP* were constructed to express the gene under control of the pdg promoter using the ATMT method^55^. Microscopic observations were performed using a Leica DM2500 fluorescent microscope with emission detected at 495–530 nm. Micrographs were examined at 1000x magnification.

### *Trichoderma* secretes cellulase in the maize root

We examined protein secretion in maize roots using immunogold electron microscopy to verify the secretion of *Thph1* or *Thph2* in maize root. The maize were inoculated with spores of the WT strain and the Δ*Thph1*, Δ*Thph2* and Δ*Thph1*&*Thph2* mutant strains along with *Curvularia lunata* (1 × 10^6^ CFU/mL) and then moisturized for 24 h, followed by surface disinfection, a potato dextrose broth (PDB) shaking culture of the root tissue for 12 h, and colloidal gold staining methods.

### Effects of the *Trichoderma Thph1* and *Thph2* genes on inhibiting lesions in maize

The seeds were pretreated with the spores from the WT strain and the maize seedlings were grown to the 4-leaf stage to study the effects of both proteins on the corn defense response. Then, the leaves were collected, washed, and introduced into flasks containing the following treatments: T1, cellulase (10 μg/ml) in water; T2, cellobiose (200 μg/ml) in water; T3, heat-inactivated cellulases; T4, cellulase + cellobiose in water; and T5, inactivated cellulase + cellobiose solution; CK, water alone. After inoculation, the leaf blades were incubated at 24 °C for 12 h, and then, all treated leaf blades were inoculated with the *Curvularia* leaf spot fungus spore suspension (1 × 10^5^ CFU/ml). The inoculated leaves were collected in a dish, to which a 1% 6-BA solution was added after wetting at 24 °C for 48 h. We measured the expression of the defense genes Opr7 and Erf1 by qRT-PCR and measured the lesion area after 72 h.

### SDS-PAGE and western blot analysis

The *Thph1* and *Thph2* genes were amplified by PCR, inserted into the pHIS2 vector, and the constructs were transformed into *E. coli* BL21 (DE3). Gene expression was induced by the addition of 1 mM IPTG. The transformants were collected, suspended in lysis buffer (50 mM NaH_2_PO_4_, 300 mM NaCl, and 10 mM imidazole), and subjected to sonication. The solution was centrifuged at 5,000 rpm for 10 min; the resulting supernatants were transferred to a Ni-NTA agarose column (Qiagen, Germany), and Thph1 and Thph2 were analyzed by immunoblotting.

A His tag was fused to the C-terminus of *Thph1* and *Thph2* to determine the interactions between the *Thph1* and *Thph2* proteins in maize root. The transferred *Thph1* or *Thph2* genes were combined with the His tag in the KO strains by ATMT. A successfully transformed mutant was obtained after screening. The stains were cultured, and the spores were coated on maize seeds.

Protein samples were electrophoresed on SDS-PAGE gels and electroblotted on a nitrocellulose membrane (Osmonics). After electroblotting, the filters were saturated with 5% non-fat dry milk in TBS with 0.1% Tween-80 for 1 h at room temperature. The *Thph1* and *Thph2* proteins were detected with specific *Thph1* and *Thph2* monoclonal antibodies tagged with a 6x His tag (dilution 1:8000; Sigma-Aldrich) using a standard western blot procedure^56^. The ECL Plus Western Blot detection reagent was used for chemiluminescent detection (GE Healthcare).

### Quantification of the maize defense-related gene expression levels

Gene-specific primers were generated for the quantitative reverse transcription RT-qPCR) analyses. PCR assays were used to determine the optimal number and optimum reaction conditions of the PCR cycles for liner amplification of the genes. At fifteen days after inoculation with the WT or KO mutant *T. harzianum* strains, the maize seedlings were inoculated with *C. lunata*. Induced systemic resistance was assayed before pathogen inoculation (CK) and in plants inoculated with the *T. harzianum* WT or KO mutant strains. Total RNA was extracted from both roots and leaves, and the cDNAs were synthesized for the RT-qPCR experiments. The mRNA expression levels were normalized to *actin* and were calculated using the 2^−ΔΔCt^ method^57^. In addition, the quantification of *Trichoderma* colonization in maize root was determined using the method reported by Li *et al*.^58^.

### Detection of the JA content by liquid chromatography-mass spectrometry (LC-MS/MS)

The JA content of fresh maize leaves was determined using the method reported by Fan *et al*.^16^. Briefly, 0.5 g of fresh maize leaves was frozen in liquid nitrogen and ground into a powder. An aliquot of 2.5 ml of 1-propanol/H_2_O-concentrated HCl (2:1:0.02 v/v/v) was added, and the samples were shaken for 30 min at 10 °C. Five milliliters of dichloromethane were also added, re-shaken for an additional 30 min, and centrifuged at 5,000 rpm for 10 min. Then, the bottom organic phase (4.5 ml) was transferred to a new 15-ml tube and evaporated under a constant nitrogen steam (approximately 20 min). Finally, each sample was re-solubilized in 2 ml of methanol, vortexed for 5 min and stored at 4 °C. The samples were injected into a 4.6 nm × 15 cm C18 5-μm column (Agilent, CA, USA). The column was maintained at 30 °C and eluted at a rate of 800 μl min^−1^ for 12 min and maintained for 5 min; the column eluent was introduced into ESI-MS (UMS ACQUITYTM UPLC & Q-TOF MS Premier). The nebulizer gas pressure, drying gas pressure, curtain gas pressure, source voltage and temperature were set to 60 psi, 50 psi, 30 kV, and 500 °C, respectively. A standard curve was prepared using methyl jasmonate (Sigma, CAS 39924-52-2) to calculate the JA content in the maize samples.

### Label-free quantitative proteomic analysis

This experiment aimed to study the effects of treatments with the *Trichoderma* WT strain (Th22) and *ΔThph1* strain on maize root proteins using label-free quantitative proteomics analysis^59^. The *Trichoderma* conidia suspensions (concentration of 1 × 10^6^ CFU/mL) were coated on the corn seeds and germinated in sterile wet filter paper for 72 h at 25 °C. Then, the germinated seeds were planted in a greenhouse pot and allowed to grow up to 4-leaf stage. The spore suspension of *Curvularia lunata* was used to inoculate the maize leaves and incubated for 48 h; then, each sample was randomly divided into three samples, and the leaves were ground into a powder in liquid nitrogen. Thirty-five milliliters of acetone-TCA (9:1) were added to the leaf powders, incubated at −20 °C overnight, and centrifuged to precipitate the proteins. The supernatant was discarded, and the precipitate was washed three times with cold pure acetone. In addition, an appropriate amount of HEPES/UA buffer (20 mM HEPES and 9 M UA, pH 7.8) was added to a weighed amount of crude protein, followed by sonication (80 W, ultrasound 10 s, intermittent 15 s, a total of 10 times), high speed centrifugation at 14,000 × *g* to obtain the supernatant, and quantification of the proteins using the Bradford method. Thirty micrograms of each sample were subjected to SDS-PAGE.

Two hundred microgram samples from each group were digested in HEPES/UA buffer to adjust the sample volume to 80 μl; then, 100 μl of a 25 mM ammonium bicarbonate solution was added. DTT was added to a final concentration of 10 mM, IAA was added to a final concentration of 50 mM, and the mixture was incubated at 37 °C for 1.5 h. Next, the sample was shaken at 600 rpm for 1 min and incubated in the dark at room temperature for 30 min, and 2 μg of Lysyl C were added and incubated at room temperature for 3 h. Four hundred microliters of 25 mM ammonium bicarbonate solution and 10 μL of trypsin buffer (20 μg of trypsin were dissolved in 50 μL of buffer) were added to the reaction system. Then, the samples were shaken (600 rpm) for 1 min, incubated at 37 °C for 16 h, and then desalted using a C18 column. One microliter of the desalinated samples was used for the MALDI-TOF mass spectrometry assay. After desalting, the lyophilized sample was subjected to peptide quantification at OD_280_ using ESI-mass spectrometry. The ESI mass spectrometry data were analyzed using SyQuest use Proteome Discoverer 1.4 software, searched using the appropriate database, and finally the results of protein evaluation were obtained. The database was searched using the downloaded UniProt sequence uniprot_Zea_mays_20150112_86964.fasta included sequence 86964 (downloaded on 2015-01-12). The filter parameters for the results were as follows: charge = 1, XCorr ≥ 1.5; charge = 2, XCorr ≥ 2.0; charge = 3, XCorr ≥ 2.25, and Delta cn < 0.1.

## Additional Information

**How to cite this article**: Saravanakumar, K. *et al*. Cellulase from *Trichoderma harzianum* interacts with roots and triggers induced systemic resistance to foliar disease in maize. *Sci. Rep*. **6**, 35543; doi: 10.1038/srep35543 (2016).

**Publisher’s note**: Springer Nature remains neutral with regard to jurisdictional claims in published maps and institutional affiliations.

## Supplementary Material

Supplementary Information
