# Supplementary material for: Cellulase from Trichoderma harzianum interacts with roots and triggers induced systemic resistance to foliar disease in maize
Source: Sci Rep. 2016 Nov 10;6:35543. doi: 10.1038/srep35543 (PMC5103226; doi:10.1038/srep35543)
Supplement: Supplementary Information [file srep35543-s1.doc]

***Supplementary Material***

**Cellulase from *Trichoderma harzianum* interacts with roots and triggers induced systemic resistance to foliar disease in maize**

Kandasamy Saravanakumar^¶^, Lili Fan^¶^, Kehe Fu, Chuanjin Yu, Meng Wang, Hai Xia, Jianan Sun, Yaqian Li, and Jie Chen^*^

^1^School of Agriculture and Biology, Shanghai Jiao Tong University, Shanghai, P.R. China

^2^State Key Laboratory of Microbial Metabolism, Shanghai Jiao Tong University, Shanghai, P.R. China

^3^Key Laboratory of Urban Agriculture (South), Ministry of Agriculture, Shanghai, P.R. China

**Competing Interests:** The authors have declared that no competing interests exist

^¶^ Shared first authorship

***Corresponding author:**

Prof. Dr. Jie Chen

Shanghai Jiao Tong University
Department of Environment and Resource, School of Agriculture and Biology
800 Dongchuan Rd
Cuddalore (Dt)
Shanghai, Shanghai 200240
China

Tel: +8621-34206141; Fax: +8621-34206141 E. mail: [jiechen59@sjtu.edu.cn](mailto:jiechen59@sjtu.edu.cn)

***Supplementary Figures***


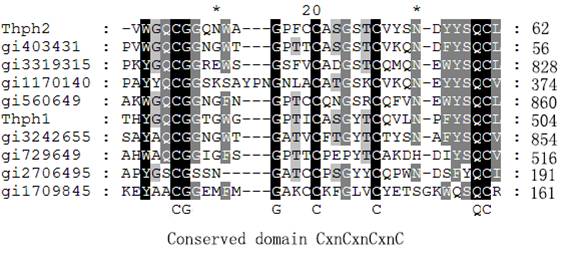


Supplementary Figure 1: NCBI blast analysis of Thph1, Thph2


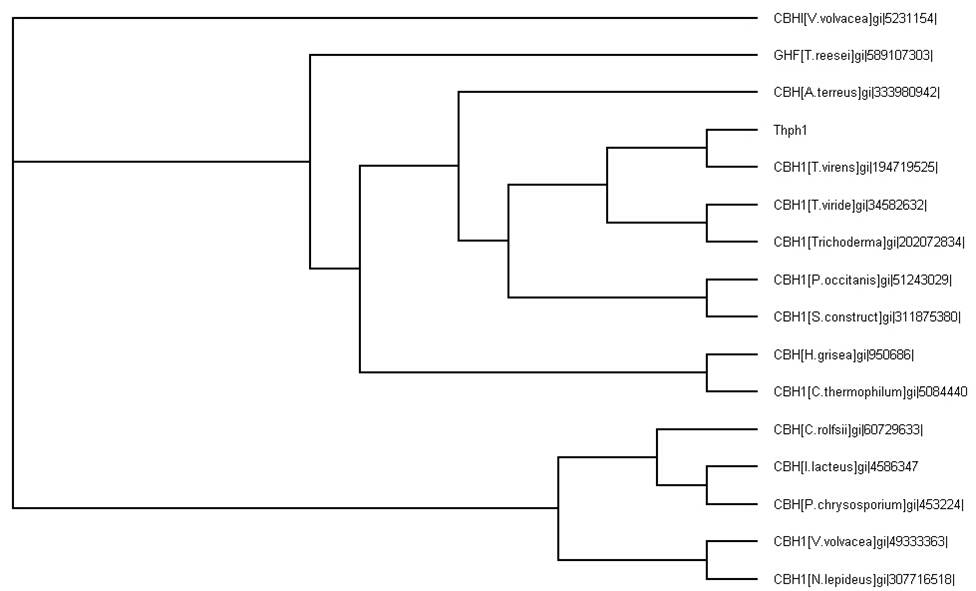


Supplementary Figure 2: Phylogeny analysis of Thph1 protein


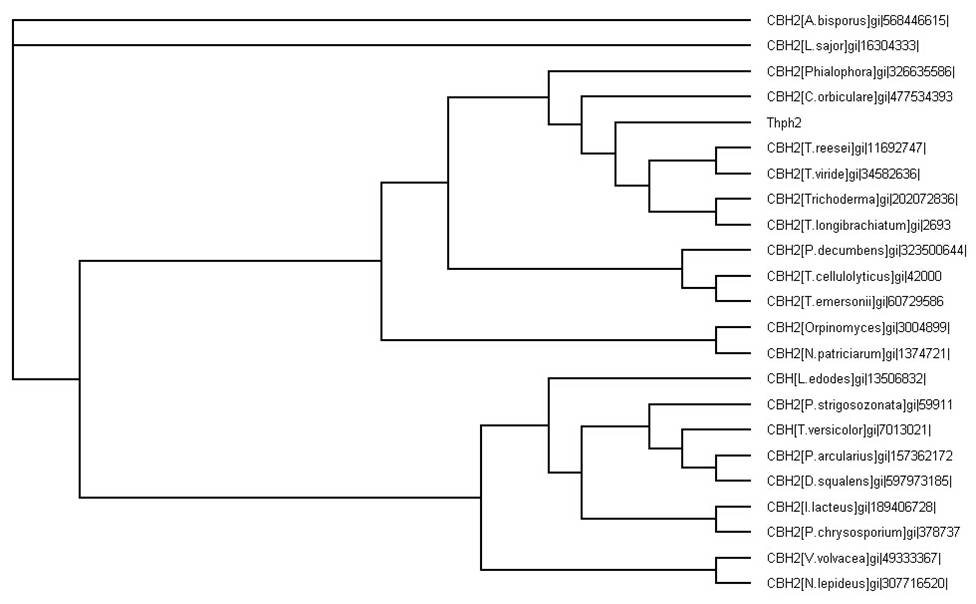


Supplementary Figure 3: Phylogeny of Thph2 protein


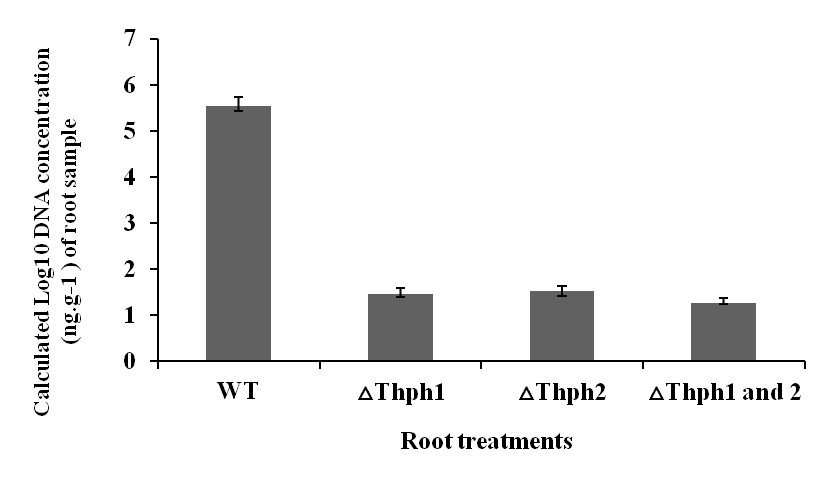
 Supplementary Figure 4: Analysis of Trichoderma WT and mutant strains colonization in maize root


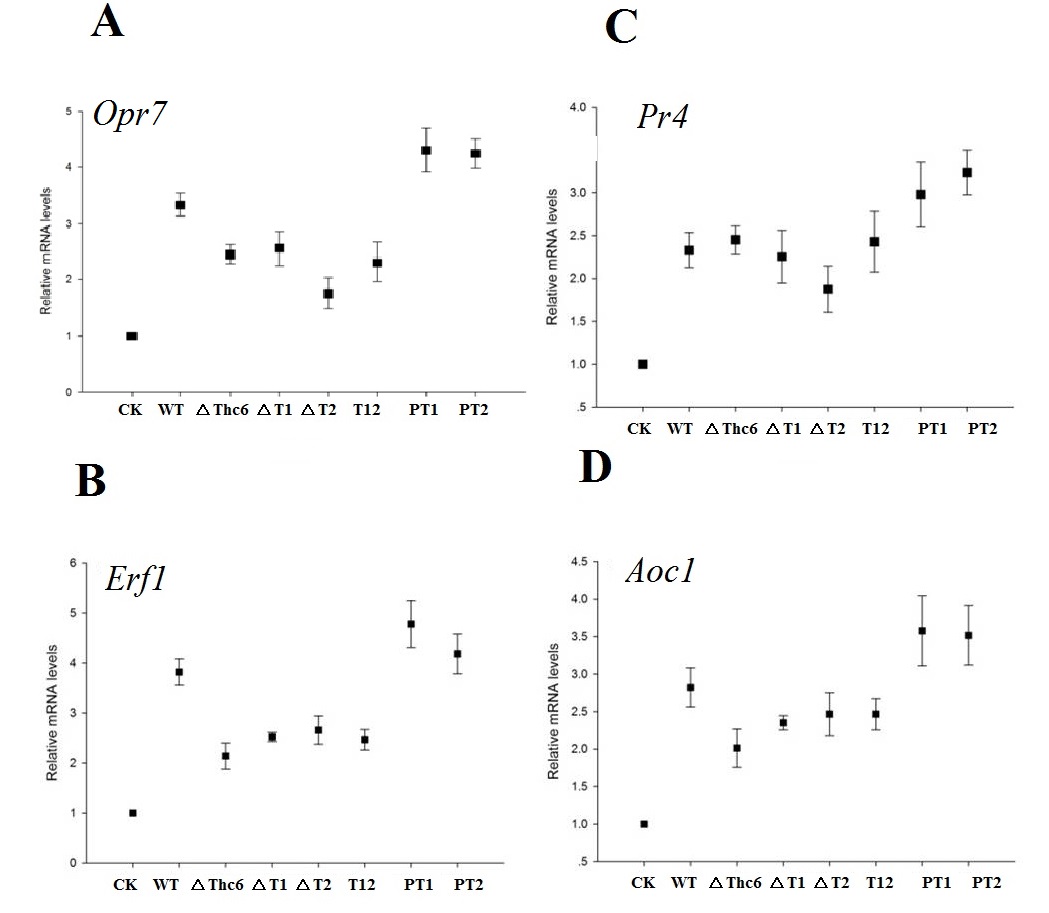


Supplementary Figure 5: qRT-PCR analysis of two defence-related gene (*Opr7*、*Pr4*、*Aoc1*、*Erf1*) expression levels in leaves of maize pretreatment with different mutants. CK, 1% sodium carboxymethyl cellulose solution. WT, wild-type strain. ΔT12, double knock-out mutant. PT1, WT+protein of Thph1. PT2, WT+protein of Thph2. Collect the sample after 24 h inoculation.


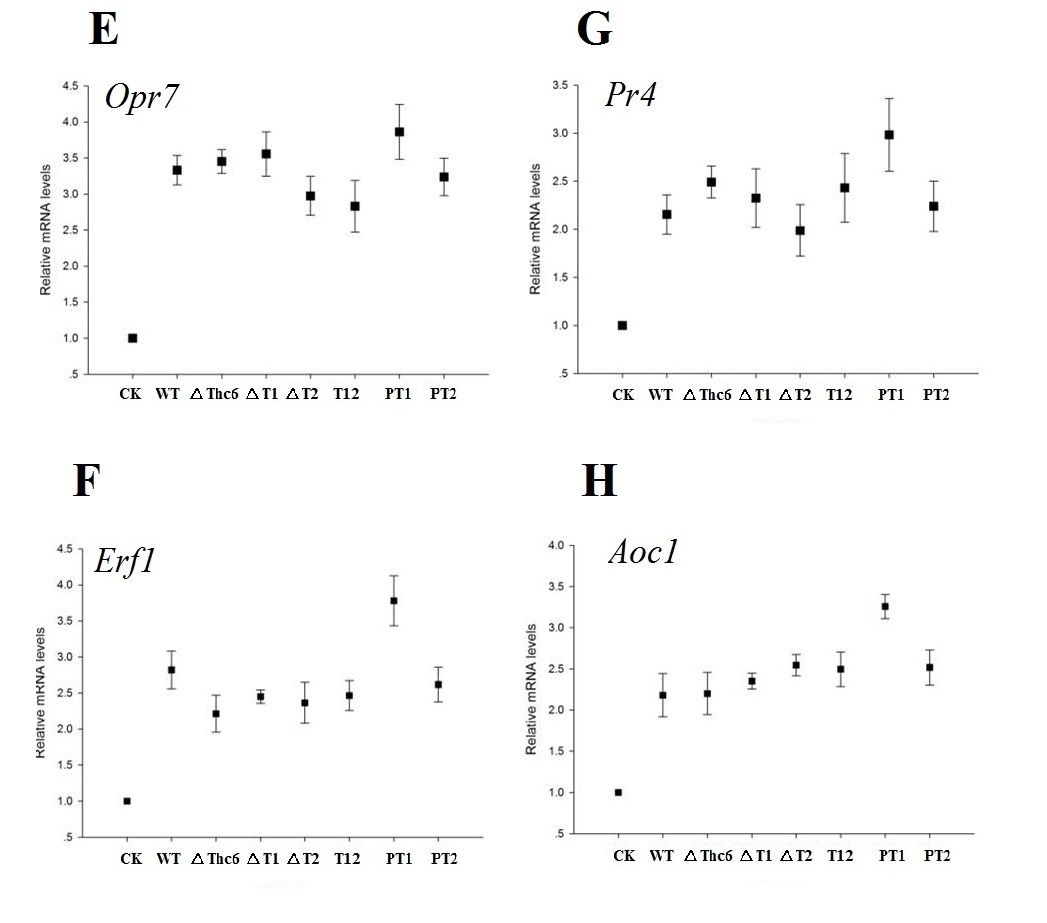


Supplementary Figure 6: qRT-PCR analysis of two defense-related gene (Opr7, Pr4, Aoc1, Erf1) expression levels in root of maize pretreatment with different mutants. CK, 1% sodium carboxymethyl cellulose solution. WT, wild-type strain. ΔT12, double knock-out mutant. PT1, WT + protein of Thph1. PT2, WT + protein of Thph2. Collect the sample after 24 h inoculation.


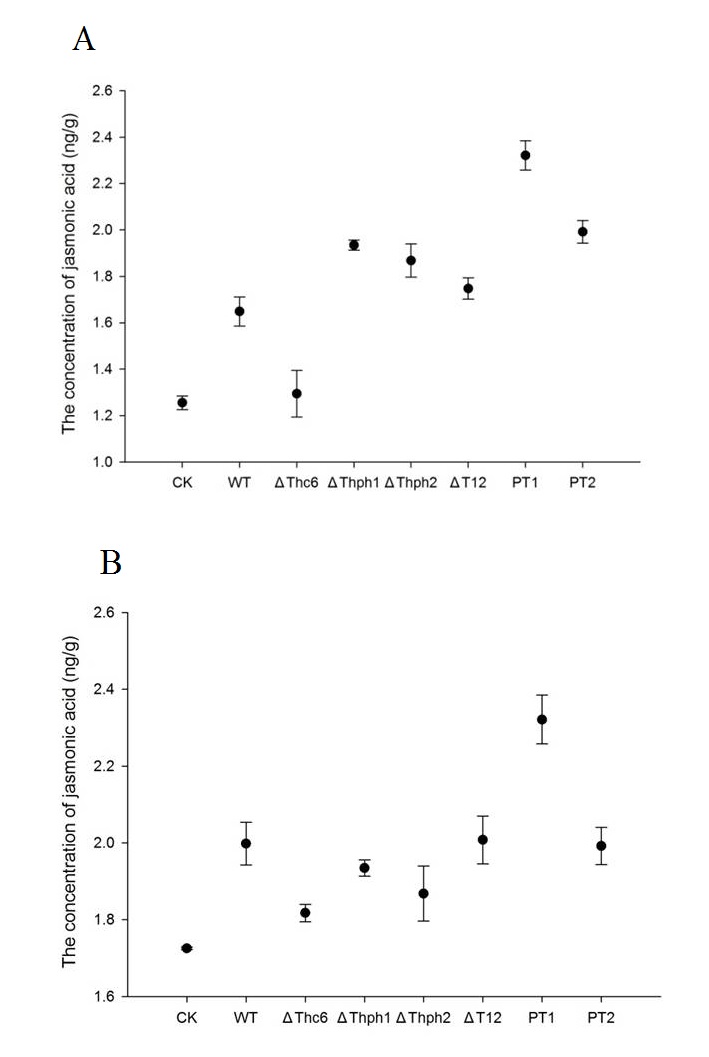


Supplementary Figure 7: Jasmonic acid concentration in (A) maize leaves and (B) maize root at different treatment of mutant and WT strains


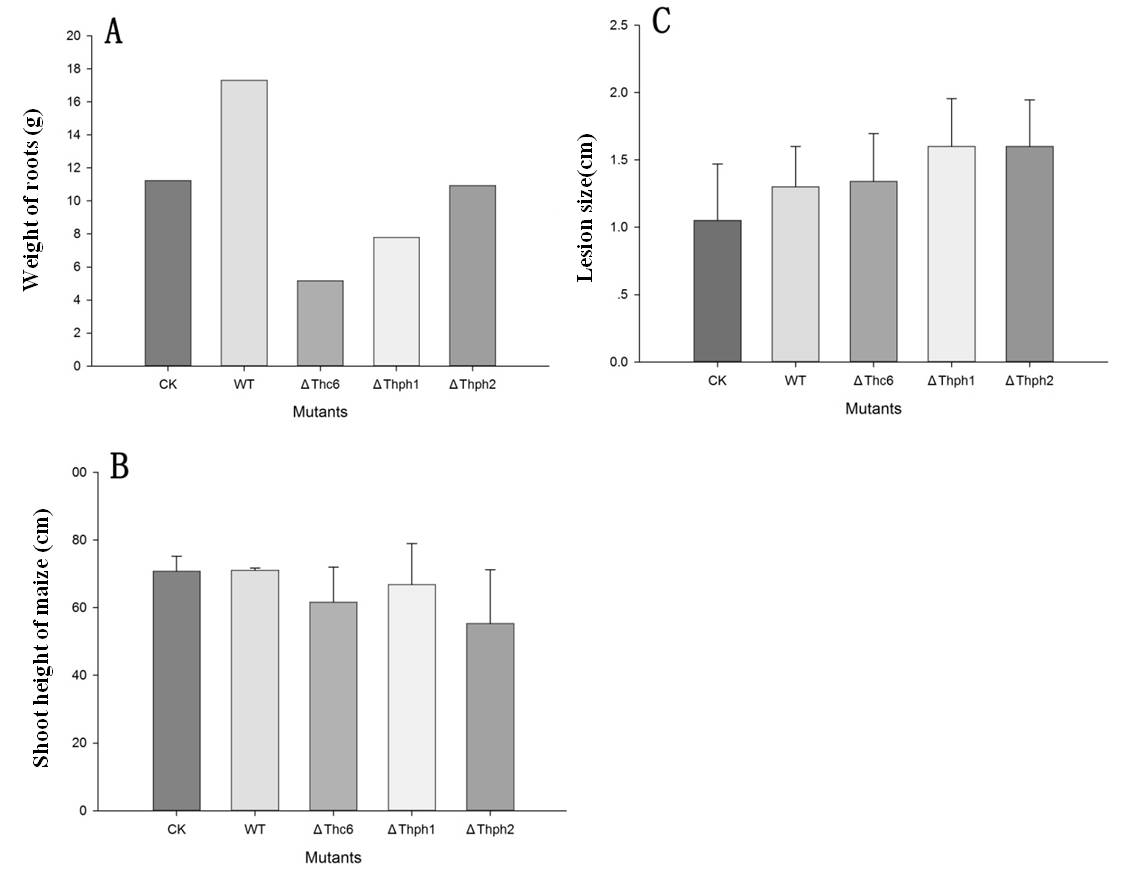


Supplementary Figure 8: Effect of KO, WT strains on weight of roots (A); shoot height of maize (B,); lesion size (C).


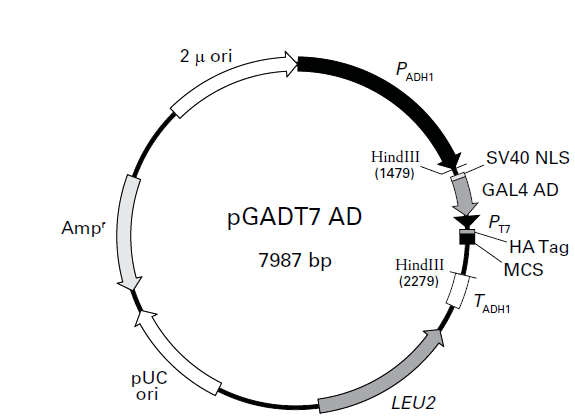


Supplementary Figure 9: Plasmid of BD with multiple cloning site (MCS)


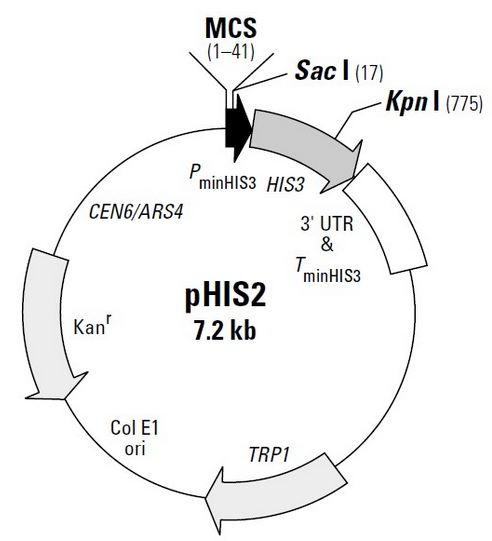


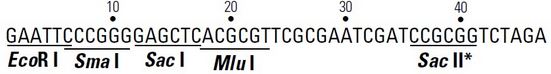


Supplementary Figure 10: Single yeast hybrid vector diagram of plasmid of pHIS2

Supplementary Table 1: Analysis of variance


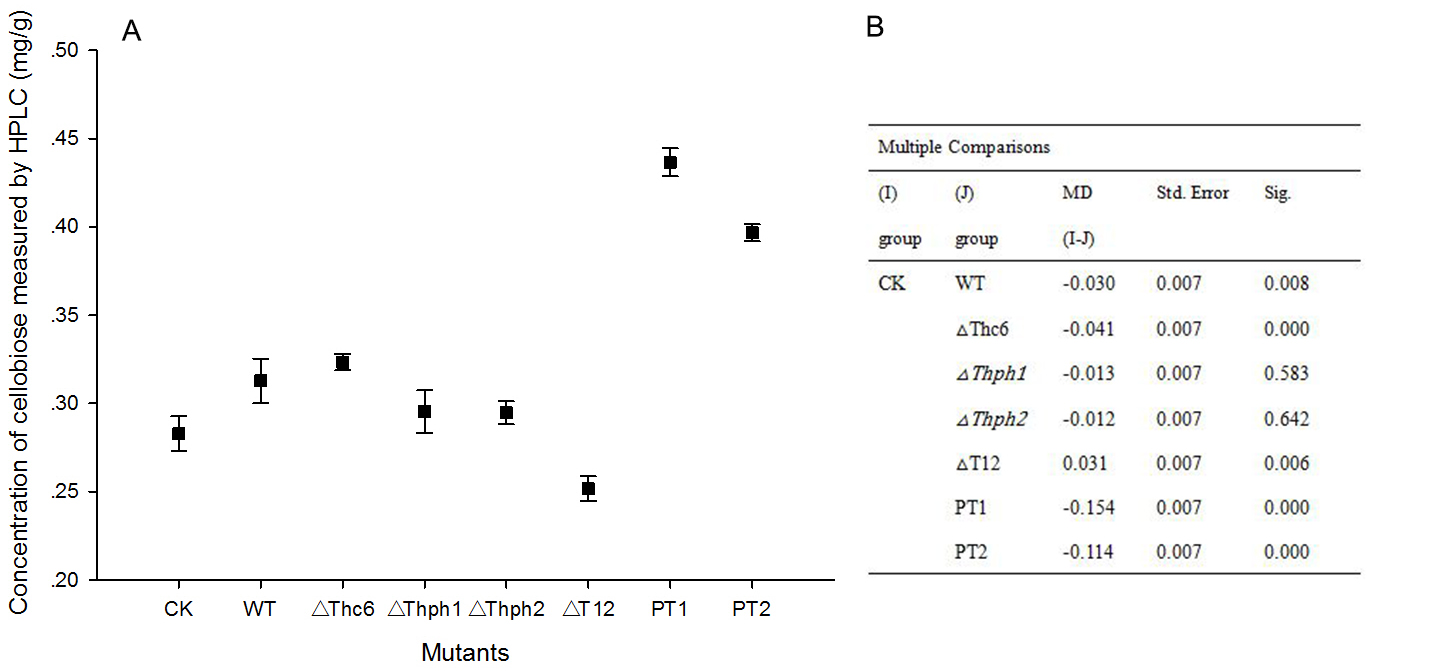


Supplementary Table 2: Effect of different time interval of *Trichoderma* treatments on root colonisations and Relative mRNA level of defence gene expressions

| Days of Trichoderma Treatments | Trichoderma DNA concentration (ng.g-1 ) | Relative mRNA level of expression | | | |
| --- | --- | --- | --- | --- | --- |
|  |  | Opr7 | Pr4 | Aoc1 | Erf1 |
| 0 | -0.22±0.01 | 0 | 0 | 0 | 0 |
| 5 | 0.77±0.23 | 0.84±0.23 | 0.68±0.06 | 0.86±0.02 | 0.30±0.06 |
| 7 | 1.26±0.12 | 0.90±0.12 | 0.80±0.03 | 1.64±0.13 | 0.96±0.10 |
| 10 | 2.50±0.11 | 1.72±0.24 | 1.57±0.06 | 1.68±0.15 | 1.22±0.14 |
| 15 | 2.91±0.08 | 2.05±0.16 | 1.83±0.23 | 1.90±0.03 | 1.02±0.08 |

Mean± SE

Supplementary Table 3: Correlation analysis of Days of treatments, Trichoderma DNA and Relative mRNA level of defence gene expressions.

|  | Days of Treatments | Trichoderma DNA (ng.g-1 ) | Relative mRNA level of expression | | | |
| --- | --- | --- | --- | --- | --- | --- |
|  |  |  | Opr7 | Pr4 | Aoc1 | Erf1 |
| Time | 1 |  |  |  |  |  |
| DNA | .976^**^ | 1 |  |  |  |  |
| Opr7 | .979^**^ | .993^**^ | 1 |  |  |  |
| Pr4 | .977^**^ | .997^**^ | .999^**^ | 1 |  |  |
| Aoc1 | .914^*^ | .916^*^ | .901^*^ | .901^*^ | 1 |  |
| Erf1 | .842 | .904^*^ | .864 | .876 | .953^*^ | 1 |

**. Correlation is significant at the 0.01 level. *. Correlation is significant at the 0.05 level.

Supplementary Table 4:OD_280_ of proteins

| sample | concentration （μg/μl） |
| --- | --- |
| 1（WT） | 1.803 |
| 2（WT） | 1.319 |
| 3（WT） | 0.621 |
| 4（*△Thph1*） | 2.734 |
| 5（*△Thph1*） | 1.370 |
| 6（*△Thph1*） | 2.286 |

Supplementary Table 5: The difference proteins identified by label-free proteomics

| sample | | The number of different proteins |
| --- | --- | --- |
| Increase  protein | Known function | 12 |
|  | Unknown and hypothetical protein | 2 |
| Down  protein | Known function | 36 |
|  | Unknown and hypothetical protein | 7 |

Supplementary Table 6: Up-regulated proteins

| Sequence number | NCBI accession number | Name of up-regulated gene | Protein ratio  (WT/△ *Thph1*) | *P value*  *(T-test*） |
| --- | --- | --- | --- | --- |
| 1 | NP_001183119 | NADP-dependent malate dehydrogenase | 7.8604 | 0.0315 |
| 2 | XP_008670327 | transcription factor MYC2-like plant myeloid tissue protein (Myelocytomatosis protein, MYCs) | 5.3491 | 0.0445 |
| 3 | NP_001132676 | protochlorophyllide reductase1 | 3.6236 | 0.0145 |
| 4 | Q9ZNY1 | Proline-rich protein precursor  (Cell wall protein) | 3.1167 | 0.0444 |
| 5 | EPS59394 | Enoyl-CoA hydratase family | 3.1010 | 0.0163 |
| 6 | NP_001151658 | 1-aminocyclopropane-1-carboxylate oxidase (ACO) | 2.6132 | 0.0042 |
| 7 | NP_001148498 | ethylene responsive protein | 2.4223 | 0.0312 |
| 8 | NP_001149630 | ethylene response protein | 2.2503 | 0.0087 |
| 9 | NP_001168118 | plant peroxidases（POD） | 2.0237 | 0.0016 |
| 10 | DAA50604 | Ferredoxin | 1.8628 | 0.0218 |
| 11 | NP_001145161 | Pyridoxine 5'-phosphate  (PNP) oxidase | 1.8130 | 0.0468 |
| 12 | NP_001146890 | HSP60（chaperonin GroEL） | 1.5159 | 0.0374 |
|  |  |  |  |  |
